# Target Optimization in Transcranial Direct Current Stimulation

**DOI:** 10.3389/fpsyt.2012.00090

**Published:** 2012-10-17

**Authors:** Rosalind J. Sadleir, Tracy D. Vannorsdall, David J. Schretlen, Barry Gordon

**Affiliations:** ^1^J. Crayton Pruitt Family Department of Biomedical Engineering, University of FloridaGainesville, FL, USA; ^2^Department of Biomedical Engineering, Kyung Hee UniversitySeoul, South Korea; ^3^Department of Psychiatry and Behavioral Sciences, The Johns Hopkins University School of MedicineBaltimore, MD, USA; ^4^Russell H. Morgan Department of Radiology and Radiological Science, The Johns Hopkins University School of MedicineBaltimore, MD, USA; ^5^Department of Neurology, Cognitive Neurology/Neuropsychology, The Johns Hopkins University School of MedicineBaltimore, MD, USA; ^6^Department of Cognitive Science, The Johns Hopkins UniversityBaltimore, MD, USA

**Keywords:** tDCS, neuroplasticity, finite element model, optimization

## Abstract

Transcranial direct current stimulation (tDCS) is an emerging neuromodulation therapy that has been experimentally determined to affect a wide range of behaviors and diseases ranging from motor, cognitive, and memory processes to depression and pain syndromes. The effects of tDCS may be inhibitory or excitatory, depending on the relative polarities of electrodes and their proximity to different brain structures. This distinction is believed to relate to the interaction of current flow with activation thresholds of different neural complexes. tDCS currents are typically applied via a single pair of large electrodes, with one (the active electrode) sited close to brain structures associated with targeted processes. To efficiently direct current toward the areas presumed related to these effects, we devised a method of steering current toward a selected area by reference to a 19-electrode montage applied to a high-resolution finite element model of the head. We used a non-linear optimization procedure to maximize mean current densities inside the left inferior frontal gyrus (IFG), while simultaneously restricting overall current, and median current densities within the accumbens. We found that a distributed current pattern could be found that would indeed direct current toward the IFG in this way, and compared it to other candidate 2-electrode configurations. Further, we found a combination of four anterior-posterior electrodes could direct current densities to the accumbens. We conclude that a similar method using multiple electrodes may be a useful means of directing current toward or away from specific brain regions and also of reducing tDCS side effects.

## Introduction

Transcranial direct current stimulation (tDCS) is an emerging method for modulation of brain function. Applications have been widely tested in experimental scenarios of motor, semantic, and attention processes (Nitsche et al., 2008). Other recent experimental uses include therapy for depression and hallucinations in schizophrenia (Brunelin et al., 2012; Loo et al., 2012).

The mechanism of tDCS is believed to arise through a modulation of baseline cortical excitability, caused by shifts in resting membrane potentials in regions experiencing current flow (Brunoni et al., 2012). The effects of tDCS depend on the relative polarity of electrodes. In general, anodal tDCS (where the active electrode is more positive than the reference electrode) has excitatory effects, and cathodal tDCS has inhibitory effects (Nitsche and Paulus, 2000). This has been substantiated in numerous experiments. For example studies of tDCS in cognitive tasks found that anodal tDCS delivered over the dorsolateral prefrontal cortex facilitated visual working memory (Fregni et al., 2005) and cathodal stimulation impaired short-term auditory memory performance (Elmer et al., 2009). Application of tDCS may, in turn affect the manifestations of neuropsychiatric conditions, including autism, depression, migraine, and schizophrenia, as baseline cortical excitability is characteristic of these conditions (Brunoni et al., 2012).

Little is known about the exact current flow patterns elicited by tDCS. Although methods using MRI scanners exist for measuring intracranial current flow (Scott et al., 1991), they are not conveniently applied because of the need for subject repositioning. Detailed models of current flow have therefore been created using finite element modeling in lieu of actual current measurement (Wagner et al., 2007; Datta et al., 2009; Sadleir et al., 2010). Though modeling is informative, there is still no clear mechanism linking current direction, current distribution, and observed experimental effects.

If it is possible to direct current toward or away from specific brain areas, the mechanisms, and structures responsible for the observed effects of tDCS may become clear. The ability to control current distribution throughout the brain may also provide a deeper understanding of general neural circuitry and networks. To best determine the stimulation parameters required to target different brain areas, we must refer to a complete electrical model of the head. This approach is natural because the paths taken by transcranial currents are defined by head geometry and conductivity, as well as electrode shape and location.

In this study, we performed tests using a non-linear optimization technique to determine if current densities in brain structures could be shaped. We investigated three scenarios: one in which we wished to target cortical structures and to avoid the accumbens; a second in which we wished to target the region of the accumbens (left and right) with no constraint on regions to be avoided; and a third in which the accumbens was targeted, but the left inferior frontal gyrus (IFG) was avoided.

Other authors have used related optimization approaches (Im et al., 2008; Dmochowski et al., 2011; Park et al., 2011). While Im et al. (2008) used a evolution strategy approach to find optimal two-electrode locations from which to target a nominated brain area, a more recent work from their group used fixed anterior and posterior electrode location and a simplex algorithm to determine the appropriate current amplitudes needed to apply maximal currents (Park et al., 2011). Similarly, Dmochowski et al. (2011) used a fixed 64-electrode array and a variety of optimization approaches to determine current amplitudes needed to create maximal currents in a nominated cortical area.

Our methods use a general non-linear algorithm, which allows for flexible and general constraints to be applied. Dmochowski et al. (2011) used a similar approach. We used a linear basis for our computations comprising calculations of current flows between individual electrodes and a reference ground plane, whereas Park et al. (2011) and Dmochowski et al. (2011) used pairs of modeled electrodes to compute test intracranial current patterns. Our approach led to the *implicit* option to include extracranial electrodes. Normally the sum of all currents flowing into and out of the head should be zero. However, in part of the work presented here we have calculated optimal current flows through electrodes without this constraint. Any uncompensated current flowing through scalp electrodes after optimization can then be accounted for in real experiments by attaching an extracranial electrode to complete the circuit and supply the remaining current. As in Dmochowski et al. (2011) we used a general non-linear algorithm that allowed the inclusion of both target and avoidance areas. In contrast to their approach, we have explicitly specified avoidance areas rather than seeking to minimize current densities in all regions outside the target. Our method used large electrodes similar to those currently used in tDCS studies. Use of large electrodes avoids the risk of applying large currents to the skin, an effect that can lead to superficial burning. Finally, the model used as the base for our computations included white matter anisotropy. This more realistic model potentially facilitates better current localization and helped us discern an intriguing anatomical asymmetry in our test model.

## Materials and Methods

In the following sections we detail our electrical head model and the constructs and calculations used in optimization procedures.

### Tissue segmentation and conductivity assignments

We used the “Re-sliced Adam” (RA) dataset from the DTI White Matter atlas repository housed at the Johns Hopkins Medical Institutes (http://cmrm.med.jhmi.edu/). The RA model is a single subject atlas with a resolution of 1 × 1 × 1 mm^3^ and includes white matter anisotropy vectors and T1 weighted (MPRAGE) MR images (Wakana et al., 2004). Segmentation was performed using both automatic classification and manual comparison with an anatomical atlas (Rubin and Safdieh, 2007). Non-brain data were segmented manually using ScanIP (Simpleware, Exeter, UK) software into 10 tissue types: cancelous bone, cortical bone, blood, cerebrospinal fluid (CSF), sclera, fat, muscle, brain, and skin. The original model did not include slices above the superior limit of the cortex. Therefore, to include the crown of the head, we extended the model by adding 12 slices (12 mm height) to the superior portion of the model, completing the head with CSF, cortical bone, and scalp materials. The brain tissue itself was further segmented automatically using FreeSurfer 5.0.0 (Cambridge, MA, USA) software into white matter and gray matter; and then subclassified into many cortical and deep brain structures. Specific target areas used in this study – the IFG, angular gyrus (AG), and dorsolateral prefrontal cortex (DLPFC) – were isolated using manually, referring anatomical atlas information.

Conductivity values were assigned to each tissue, chosen from measurements reported below 1 kHz. Table 1 lists the sources for conductivities.

**Table 1:** **Conductivities assigned to tissues in our model**.

Compartment	Conductivity (S/m)	Reference
Air	0	–
Skin	4.3 × 10^−1^	Holdefer et al. (2006)
Cerebrospinal fluid	1.8 × 10^0^	Baumann et al. (1997)
Sclera	5.0 × 10^−1^	Gabriel et al. (1996)
Cortical bone	5.52 × 10^−3^	Akhtari et al. (2002)
Cancelous bone	21.4 × 10^−3^	Akhtari et al. (2002)
Muscle*	1.6 × 10^−1^	Geddes and Baker (1967)
Fat	2.5 × 10^−2^	Gabriel et al. (1996)
Blood	6.7 × 10^−1^	Geddes and Baker (1967)
White matter*	1.2 × 10^−1^ (trans.)	Geddes and Baker (1967)
	1.2 × 10^−0^ (long.)	
Gray matter	1.0 × 10^−1^	Gabriel et al. (1996)

Values were chosen from available low frequency (<1 kHz) data in the literature. Compartments marked with an asterisk were anisotropic. The isotropic conductivity assigned to muscle was calculated according to the formula σ* = (σ*_l_* σ_t_) where σ*_l_* is longitudinal and σ*_t_* is transverse measured conductivity.

White matter was assumed anisotropic. We distinguished between conductivities of cancelous and cortical bone because of the large electrical property differences between these tissues (Akhtari et al., 2000, 2002; Sadleir and Argibay, 2007).

### Finite element modeling

The model solved the Laplace equation

(1) $$\begin{array}{c} \nabla \cdot \left(\sigma \left(x,y,z\right)\;\nabla \phi \right)=0 \end{array}$$

on the domain Ω (the head), subject to

(2) $$\begin{array}{c} \sigma \frac{\partial \phi }{dn}=j\text{ and }\phi_{\text{base}}\text{ = 0;} \end{array}$$

on the surface of the domain *d*Ω. Here, σ(*x*,*y*,*z*) is the conductivity distribution within the head, Φ is the voltage distribution, *j* is the surface current density, and **n** is a vector normal to the surface. The quantity *j* was only non-zero on electrodes. The voltage on the base plane (the caudal slice) of the model (Φ_base_) was set to zero.

The segmented phantom was converted into a quadratic tetrahedral finite element model containing ∼18 million elements. In each white matter voxel, the anisotropic conductivity tensor was calculated as

$${\text{D}}_{W}={\text{A}}^{T}{{\text{D}}^{*}}_{W}\text{A}$$

where

${{\text{D}}^{*}}_{W}=\left[\begin{array}{ccc} \sigma_{l} & 0 & 0\\ 0 & \sigma_{t} & 0\\ 0 & 0 & \sigma_{t} \end{array}\right]$ and ***A** *= ***R**_z_**R**_y_**R**_x_*. ***R**_z_*, ***R**_y_*, and ***R**_x_* are rotation matrices about the *z*, *y*, and *x* axes, respectively. In isotropic voxels, **D** was a diagonal matrix with all entries equal to the local isotropic conductivity value.

Computations of finite element model matrix equations and boundary conditions were implemented in C and solved using the preconditioned conjugate gradient method.

#### Electrode assignment and definition

**Figure 1:**
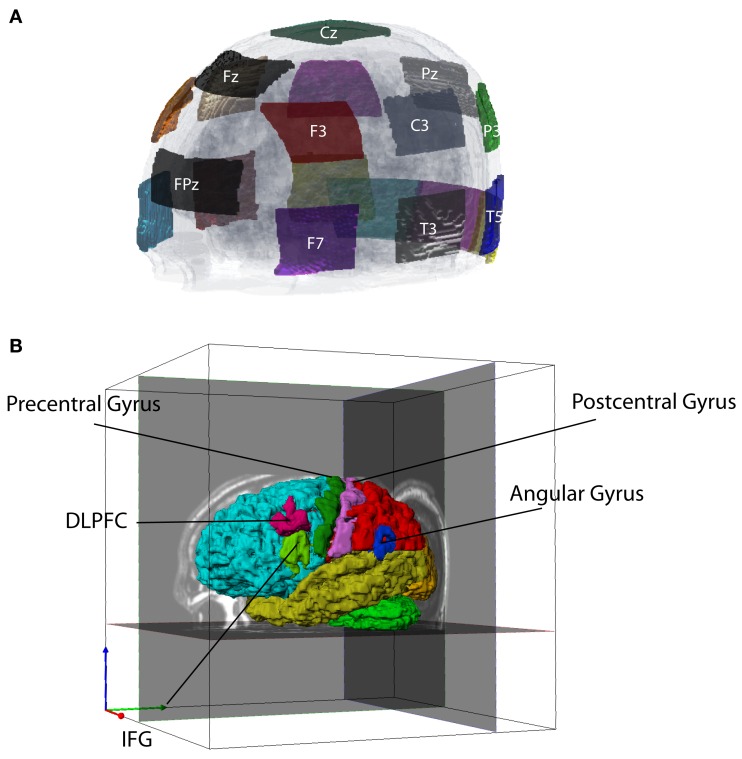
**Electrode montage and tissue segmentation**. **(A)** Left frontal view of electrode montage shown on a transparent head model. There were 19 electrodes in total, not all of which are shown. **(B)** Segmented cortical structures, showing frontal (aqua), parietal (red), temporal (yellow), occipital (orange) lobes, cereballar gray matter (green), angular gyrus (AG, blue), dorsolateral prefrontal cortex (DLPFC, cyan), inferior frontal gyrus (IFG, light green), precentral cortex (dark green), and postcentral cortex (pink).

Transcranial direct current stimulation current is normally introduced via a pair of large (∼35 cm^2^) saline/sponge electrodes. One (the active electrode) is sited close to brain regions presumed involved in target processes. The other (the reference electrode) is placed elsewhere on the head or body. For this study, we defined a montage of NE = 19 electrodes (Figure 1). The electrodes were selected from standard 10–20 EEG locations. Each electrode had an area of ∼22 cm^2^. Use of large electrodes reduces the risk that superficial burns will result from current application.

#### Boundary conditions

**Figure 2:**
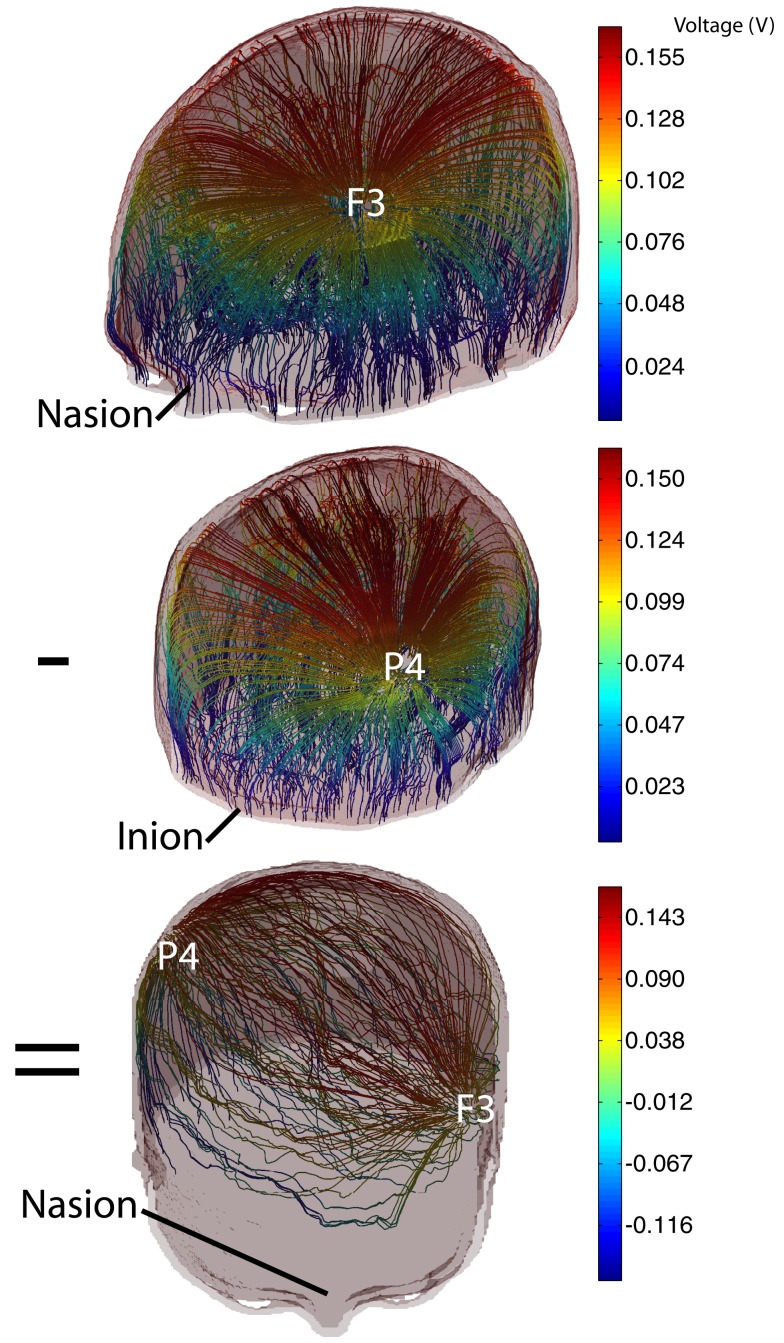
**Illustration of addition of 2 base voltage distributions with opposite weights to form a generic 2-electrode tDCS pattern**. (Top) Current streamlines formed between the electrode at F3 and model base; (center) current streamlines formed between P4 and model base; (bottom) current streamlines resulting from subtraction of P4 data from F3 data. Streamline colors are indexed to voltage values.

The base data used in the optimization procedure consisted of voltage data calculated between each electrode and a ground plane situated at the base of the model (Figure 2). In calculating the voltage data for each isolated electrode in turn, we simulated a total current of 1 mA injected into the head. Use of this single electrode arrangement allowed us to include the possibility that extracranial electrodes could be included (simply by allowing the sum of currents applied to the model to have a net non-zero value, implying that the extra current flowed through the neck and to an electrode located away from the head.

### Data computation

Voltage distributions for a particular electrode combination were computed using the principle of superposition by summing the weighted basis data set as

(3) $$\begin{array}{c} V_{X}=X_{1}V_{1}+X_{2}V_{2}+\cdots +X_{\text{NE}}V_{\text{NE}} \end{array}$$

where ***X*** = [*X*_1_*X*_2_…*X_NE_*] was a vector of weighting factors for each voltage data set, *V*_1 …*NE*_ were the basis data sets, and *V_X_* was the resulting voltage. Figure 2 shows the result of weighted summation of voltage basis data using electrodes F3 and P4. The top and center panels of Figure 2 show individual voltage basis data for electrodes F3 and P4, and the lower panel shows the result of adding data for electrode F3 (weight 1 mA) to data for P3 (weight −1 mA). The total current magnitude injected into the head was computed as

(4) $$\begin{array}{c} C_{\text{total}}=\frac{1}{2}\overset{NE}{\underset{i=1}{\sum }}\left|X_{i}\right| \end{array}$$

The current density **J** in each voxel *k* was calculated as

(5) $$\begin{array}{c} J_{k}=-{\text{D}}_{k}\nabla \phi \end{array}$$

where ▽Φ is the local voltage gradient.

Current density norms *J* were calculated within each voxel from individual vector components as

(6) $$\begin{array}{c} J={\left(J_{x}^{2}+J_{y}^{2}+J_{z}^{2}\right)}^{1/2} \end{array}$$

This distribution was then used to compute mean or median current densities within regions of interest.

### Optimization procedure

We used the interior point optimization method to calculate the optimal electrode currents. The interior point algorithm (Waltz et al., 2006) solves a general non-linear minimization problem subject to linear and non-linear constraints. Other methods for solving such problems include sequential quadratic programming methods (Bonnans et al., 2006) and simulated annealing (Kirkpatrick et al., 1983).

Our interior point optimization algorithm was implemented in the MATLAB (Natick, MA, USA) function fmincon to solve.

$$\begin{array}{llll} & \underset{X}{\max}\left[\text{mean}\left(J_{\text{target}}\left(\text{X}\right)\right)\right],\; & & \\ & \text{such that }\left\{\;\begin{array}{cc} \overset{NE}{\underset{i=1}{\sum }}X_{i}=0 & \left(1\right)\\ \text{mean}\left(J_{\text{avoid}}\left(\text{X}\right)\right)<J_{\max} & \left(2\right)\\ \overset{NE}{\underset{i=1}{\sum }}\left|X_{i}\right|>C_{\min} & \left(3\right)\\ \overset{NE}{\underset{i=1}{\sum }}\left|X_{i}\right|<C_{\max} & \left(4\right)\\ \text{mean}\left(J_{\text{target}}\left(\text{X}\right)\right)\geq r\;\text{mean}\left(J_{\text{avoid}}\left(\text{X}\right)\right) & \left(5\right) \end{array}\right. & & \text{(7)} \end{array}$$

Here, ***X*** is the vector consisting of coefficients denoting the stimulus intensity to be delivered to each electrode, and *J* refers to the current density norm within a brain structure (a *target* region or a region to *avoid*). The quantity $\underset{X}{\max}\left[\text{mean}\left(J_{\text{target}}\left(\text{X}\right)\right)\right]$ is the objective function. The optimization is subject to the constraints that the total current injected into the brain is zero (constraint 1), the mean *J* delivered to the “avoid” region is less than a prescribed maximum value (*J*_max_, constraint 2), the total absolute delivered current is above a set threshold (*C*_min_, constraint 3) and below another threshold (*C*_max_, constraint 4), and the mean *J* in the target region is at least *r* times the mean current density in the avoid region, where *r* is a dimensionless constant (constraint 5). Only constraint 4 is essential. For example, if constraint 1 is not applied, any unbalanced flow of current through the ground plane may be considered as flow to or from an extracranial electrode, such as those used in several previous studies (Cogiamanian et al., 2007; Monti et al., 2008; Priori et al., 2008). We may consider other constraints, such as a limit on the maximum skin *J*.

#### Termination criteria

The optimization procedure was terminated if more than 100 iterations were required, if the relative step size of any iteration was below 1 part in 10^10^ or if the gradient estimate was below 1 part in 10^3^. A feasible solution was considered achieved if the maximum constraint violation was smaller than 1 part in 10^10^_._

#### Mean and median current density values

Although we have previously (Sadleir et al., 2010) quoted median current densities as best representative of distributions, and have observed that the current density distributions are approximately log-normal, there is no analytical method to associate the median of sums and the sum of medians for log-normal distributions (Limpert et al., 2001). This limitation prevents us from associating median current densities in individual base current distributions with the median of their sum. Consequently, the gradient of the objective function cannot be computed, except numerically. Numerical gradient estimation requires many extra function estimations and greatly slows the optimization algorithm. We therefore estimated the gradient of the objective function by computing the mean *J* created in the target region for each of the 19 candidate patterns. This approach does not produce an exact gradient, but the sum of weighted mean current densities is greater than or equal to actual mean *J* values, that is

$$\begin{array}{llll} & J=\left|\text{J}\right|\leq X_{1}\left|{\text{J}}_{1}\right|+X_{2}\left|{\text{J}}_{2}\right|+\cdots +X_{NE}\left|{\text{J}}_{NE}\right|, & & \\ & J_{\text{target},\text{avoid}}=\left|{\text{J}}_{\text{target},\text{avoid}}\right|\leq X_{1}\left|{\text{J}}_{1}^{\text{target},\text{avoid}}\right|+X_{2}\left|{\text{J}}_{2}^{\text{target},\text{avoid}}\right| & & \\ & +\cdots +X_{NE}\left|{\text{J}}_{NE}^{\text{target},\text{avoid}}\right|\text{ and} & & \\ & \text{mean}\left(J\right)\leq \text{mean}\left(X_{1}\left|{\text{J}}_{1}\right|\right)+\text{mean}\left(X_{2}\left|{\text{J}}_{2}\right|\right)+\cdots & & \\ & +\text{mean}\left(X_{NE}\left|{\text{J}}_{NE}\right|\right)\text{ or} & & \\ & \text{mean}\left(J_{\text{target},\text{avoid}}\right)\leq \text{mean}\left(X_{1}\left|{\text{J}}_{1}^{\text{target},\text{avoid}}\right|\right)+\text{mean} & & \\ & \left(X_{2}\left|{\text{J}}_{2}^{\text{target},\text{avoid}}\right|\right)+\cdots +\text{mean}\left(X_{NE}\left|{\text{J}}_{NE}^{\text{target},\text{avoid}}\right|\right). & & \text{(8)} \end{array}$$

In the first step of the optimization algorithm, our precomputed gradient was compared with internal estimations of gradient and found to agree within a relative tolerance of 1 × 10^−6^. Thus, we believe that the precomputed gradient provided a satisfactory estimate to guide optimization. In the results that follow, we continue to present our findings in terms of median values.

### Problems considered

We tested the optimization procedure in the context of three different problems. First, we sought to deliver current preferentially to the left IFG, while avoiding delivery to the accumbens (Problem 1). In this problem we required that the *J*_max_ experienced by left and right accumbens was less than 0.5 μA/cm^2^, while we chose *C*_min_ and *C*_max_ to be 0.5 and 2 mA, respectively. We also required that the mean *J* in the left IFG was at least twice the mean *J* in the accumbens (*r* = 2).

In Problem 2, we wished to deliver maximal *J* to the accumbens. No “avoid” region was nominated, but we again chose *C*_min_ and *C*_max_ to be 0.5 and 2 mA, respectively.

In Problem 3, we again nominated the accumbens as the target, but specified that the left IFG be avoided. We set the mean *J* ratio, *r*, to be 1. Again, *C*_min_ and *C*_max_ were 0.5 and 2 mA, respectively.

## Results

### Problem 1

**Figure 3:**
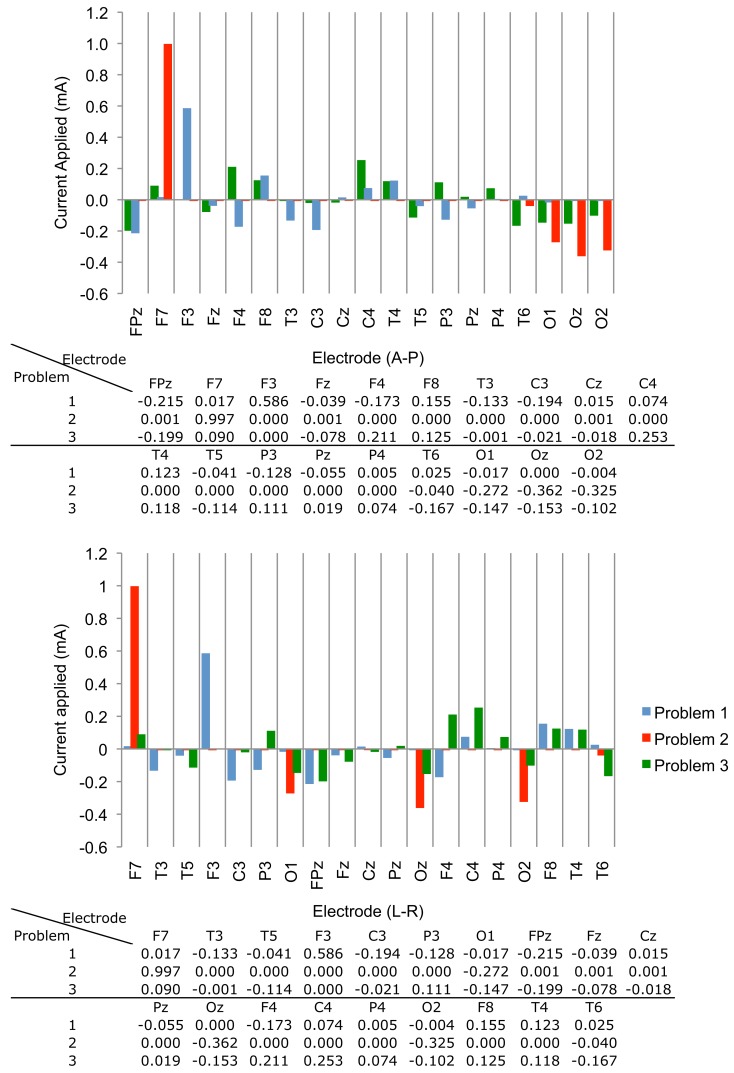
**Optimized current weights (in mA) found in the three problems, shown in graphical and tabular format**. Weights are displayed in (top) Anterior-Posterior and (bottom) Left-Right arrangements.

We executed Problem 1 using the procedures outlined above and obtained a result **X** that satisfied all constraints. The optimization algorithm was terminated because the step size was smaller than the threshold value of 10^−10^. First-order optimality was found to be around 10^−3^. The values of individual coefficients are plotted in Figure 3. Note that the positive weights of each electrode were biased toward those near the left IFG, such as F3.

**Figure 4:**
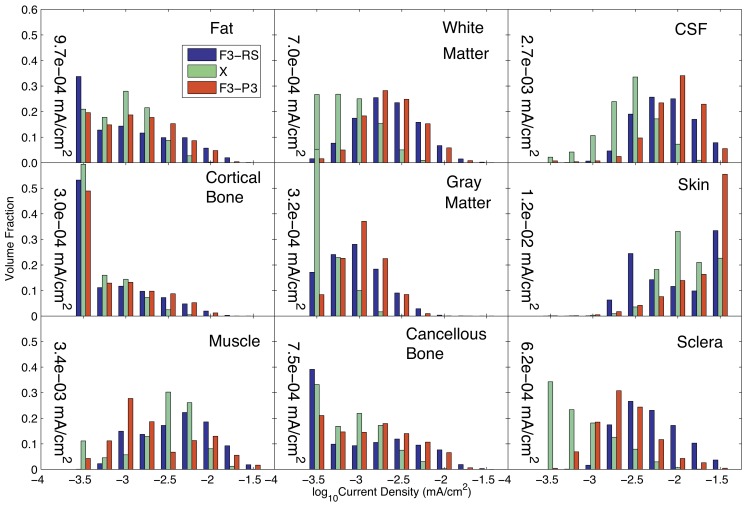
**Comparison of current density distributions formed by three candidate current patterns (the optimal solution to Problem 1, X; a configuration previously used in the literature (F3-RS); and a configuration formed using the two electrodes with the largest weights of X**. Median values found in each tissue for the optimal solution**(X)** are shown within each graph of the figure.

We compared the results of Problem 1 optimization with those achieved for an earlier simulation in which only two electrodes were used [F3 and a right supraorbital (RS) electrode]. We also computed the current densities resulting from an F3-P3 pattern, given that the estimated**X** value contained large coefficients for each of these electrodes. The results for these three configurations are compared in Figure 4, showing the current distributions in different tissues. The 1-norm of the total current found for our “optimized” problem, *C* = 1.15 mA, was scaled so that the total injected current had the same value of 1 mA in all three configurations.

The median current density in different tissues found in each of these three configurations is shown in Table 2. The current densities in the target and avoided regions are highlighted in green and red respectively.

**Table 2:** **Median current density values found in different tissues and structures for Problem 1**.

	**X**	F3-RS	F3-P3
	mA/cm^2^	mA/cm^2^	mA/cm^2^
**TISSUE**			
Blood	1.04 × 10^−4^	4.45 × 10^−3^	4.85 × 10^−4^
Cancelous bone	7.49 × 10^−5^	8.04 × 10^−4^	1.32 × 10^−4^
Cortical bone	2.97 × 10^−5^	3.66 × 10^−4^	4.41 × 10^−5^
CSF	2.75 × 10^−4^	7.49 × 10^−3^	9.36 × 10^−4^
Fat	9.67 × 10^−4^	8.54 × 10^−4^	1.22 × 10^−4^
Gray matter	3.21 × 10^−4^	9.06 × 10^−4^	1.01 × 10^−4^
Muscle	3.36 × 10^−3^	4.52 × 10^−3^	1.53 × 10^−4^
Sclera	6.22 × 10^−4^	4.65 × 10^−3^	2.12 × 10^−4^
Skin	1.18 × 10^−2^	9.42 × 10^−3^	2.80 × 10^−3^
White matter	6.98 × 10^−4^	2.26 × 10^−3^	2.22 × 10^−4^
**CORTICAL STRUCTURE**			
AG (L)	7.20 × 10^−4^	9.31 × 10^−4^	2.22 × 10^−3^
AG (R)	3.10 × 10^−4^	5.96 × 10^−4^	7.00 × 10^−4^
Cingulate	2.88 × 10^−4^	9.09 × 10^−4^	1.18 × 10^−3^
DLPFC (L)	1.13 × 10^−3^	1.52 × 10^−3^	2.82 × 10^−3^
DLPFC (R)	3.26 × 10^−4^	1.68 × 10^−3^	9.78 × 10^−4^
Frontal lobe	4.39 × 10^−4^	2.07 × 10^−3^	1.31 × 10^−3^
IFG (L)	8.26 × 10^−4^	1.87 × 10^−3^	2.14 × 10^−3^
IFG (R)	2.63 × 10^−4^	1.49 × 10^−3^	7.73 × 10^−4^
Occipital lobe	3.14 × 10^−4^	3.84 × 10^−4^	8.89 × 10^−4^
Parietal lobe	3.92 × 10^−4^	6.45 × 10^−4^	1.20 × 10^−3^
Temporal lobe	3.49 × 10^−4^	8.72 × 10^−4^	8.93 × 10^−4^
**DEEP STRUCTURE**			
Accumbens	9.74 × 10^−5^	1.38 × 10^−3^	9.08 × 10^−4^
Amygdala	2.19 × 10^−4^	1.37 × 10^−3^	1.18 × 10^−3^
Caudate nucleus	1.66 × 10^−4^	1.70 × 10^−3^	9.13 × 10^−4^
Cerebellar GM	2.03 × 10^−4^	4.06 × 10^−4^	4.92 × 10^−4^
Hippocampus	2.46 × 10^−4^	1.15 × 10^−3^	8.91 × 10^−4^
Globus pallidus	1.65 × 10^−4^	1.20 × 10^−3^	9.26 × 10^−4^
Putamen	2.06 × 10^−4^	1.37 × 10^−3^	8.70 × 10^−4^
Thalamus	2.01 × 10^−4^	1.03 × 10^−3^	1.02 × 10^−3^

*The optimal weighting **X** was compared with another candidate pattern (F3-RS) and a pattern found using the 2 greatest weights in **X**. Median values in targeted and avoided regions are highlighted in green and red shading, respectively*.

**Figure 5:**
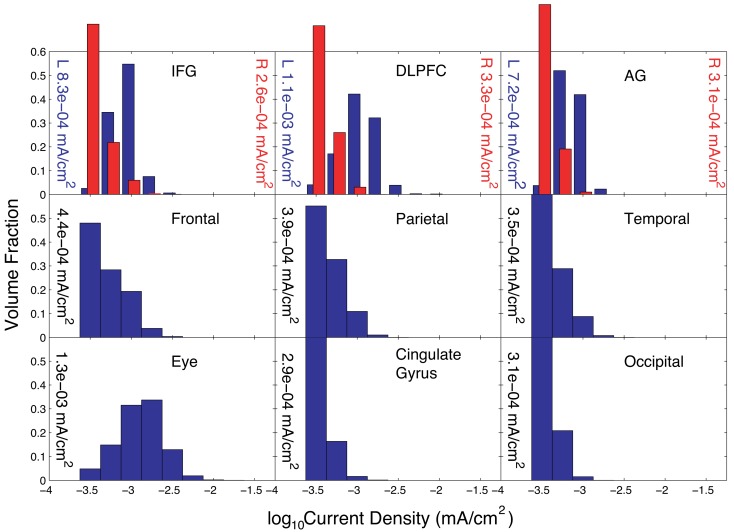
**Distribution of current densities in peripheral cortical structures in Problem 1 found using the optimal solution, X**. Median values in each structure obtained using the optimal solution are shown within each graph of the figure. IFG, DLPFC, and AG refer to the inferior frontal gyrus (IFG), dorsolateral prefrontal cortex (DLPFC), and angular gyrus (AG), respectively. For the IFG, DLPFC, and AG, current density distributions are shown separately for left (blue) and right (red) structures, with median values shown on either side of each plot.

The current distributions in peripheral cortical tissues are summarized in Figure 5. The distributions in the IFG, DLPFC, and angular gyrus are shown bilaterally. The median current densities in the left IFG were approximately four times those in the right IFG.

### Problem 2

Solution of Problem 2, which sought to maximize mean current densities in the accumbens with no “avoid” region specified, was terminated because the maximum number of iterations was exceeded. However, substantial progress toward a solution was made. We found that the optimization procedure produced a clear bias toward anterior and posterior electrodes. Also, there were only four electrodes with an absolute normalized weight greater than 1 μA – electrodes F7, O1, O2, Oz, and T6. The electrode with the largest weight, F7, was not centrally located, being on the lower left head, and all other electrodes had negative weights. We believe that this unexpected bias may have resulted from inhomogeneity in the conductivity distribution or white matter directions. The problem resulted in a first-order optimization value of about 2 × 10^−3^, larger than the value found in solving Problem 1.

**Figure 6:**
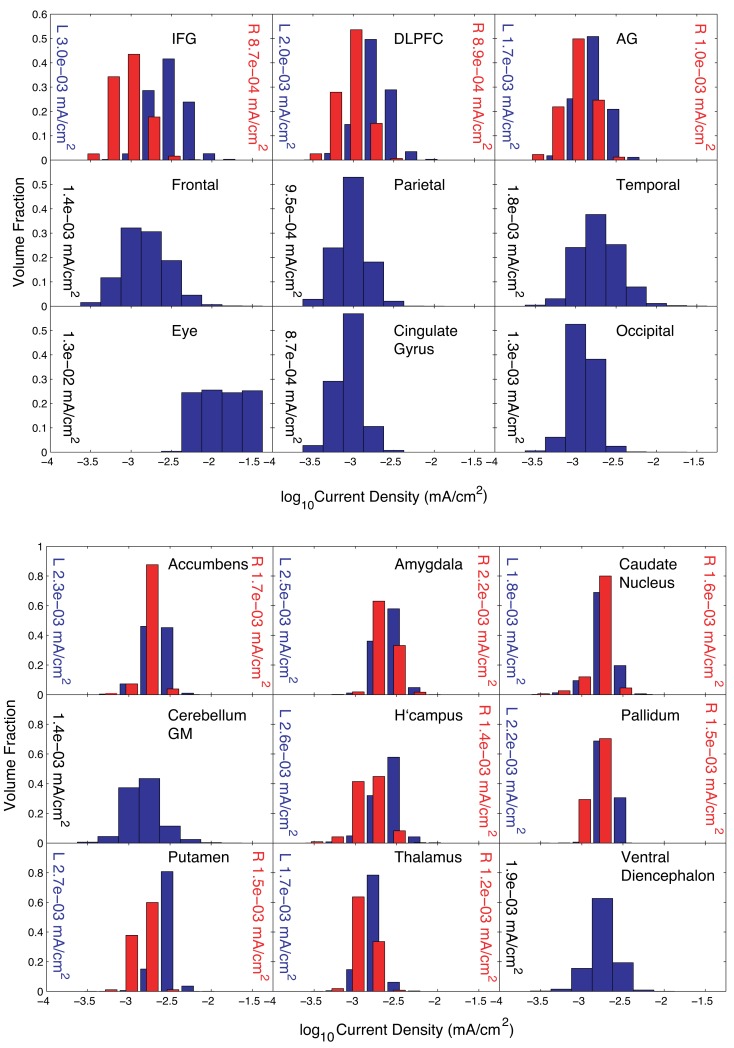
**Distribution of current densities in (top) peripheral cortical structures and (bottom) deep brain structures for Problem 2**. Median values in each structure obtained using the optimal solution **X** are shown within each graph of the figure. IFG, DLPFC, and AG refer to the inferior frontal gyrus (IFG), dorsolateral prefrontal cortex (DLPFC), and angular gyrus (AG), respectively. For the IFG, DLPFC, and AG, current density distributions are shown separately for left (blue) and right (red) structures, with median values shown on either side of each plot.

Distribution estimations within basal ganglia and peripheral cortical structures for Problem 2 are plotted in Figure 6 for the normalized optimized pattern. A comparison with a 2-electrode pattern chosen by using only the electrodes with the two largest magnitude weights found by the optimization procedure, F7 and Oz, is shown in Table 3. The current densities found in target structures by the optimization procedure (using five electrodes) were very similar to this 2-electrode pattern. Median eye current densities found for both the F7-Oz pattern and the optimal solution were around 10 μA/cm^2^. The threshold for phosphene generation cited in the literature (8 mA/m^2^ or 0.8 μA/cm^2^; Reilly, 1998) was based on stimulation at 20 Hz. Therefore, even though the threshold for DC stimulation might in fact be at least a factor of 10 higher (Adrian, 1977), we would expect this current pattern to produce phosphenes.

**Table 3:** **Median current density values found in different tissues and structures for Problem 2**.

	**X**	FPz-Oz	F7-Oz
	mA/cm^2^	mA/cm^2^	mA/cm^2^
**TISSUE**			
Blood	6.00 × 10^−3^	5.94 × 10^−3^	6.07 × 10^−3^
Cancelous bone	6.89 × 10^−4^	6.69 × 10^−4^	6.81 × 10^−4^
Cortical bone	6.09 × 10^−4^	3.42 × 10^−4^	5.76 × 10^−4^
CSF	1.00 × 10^−2^	1.21 × 10^−2^	1.02 × 10^−2^
Fat	1.09 × 10^−3^	8.46 × 10^−4^	9.90 × 10^−4^
Gray matter	1.38 × 10^−3^	1.36 × 10^−3^	1.40 × 10^−3^
Muscle	6.84 × 10^−3^	3.56 × 10^−3^	6.78 × 10^−3^
Sclera	7.47 × 10^−3^	6.58 × 10^−3^	7.53 × 10^−3^
Skin	1.14 × 10^−2^	8.38 × 10^−3^	1.11 × 10^−2^
White matter	6.92 × 10^−3^	2.81 × 10^−3^	6.96 × 10^−3^
**CORTICAL STRUCTURE**			
AG (L)	1.69 × 10^−3^	1.27 × 10^−3^	1.70 × 10^−3^
AG (R)	1.00 × 10^−3^	1.27 × 10^−3^	9.80 × 10^−4^
Cingulate	8.70 × 10^−4^	1.07 × 10^−3^	8.86 × 10^−4^
DLPFC (L)	1.99 × 10^−3^	1.86 × 10^−3^	2.00 × 10^−3^
DLPFC (R)	8.92 × 10^−4^	1.71 × 10^−3^	8.92 × 10^−4^
Frontal lobe	1.44 × 10^−3^	1.82 × 10^−3^	1.45 × 10^−3^
IFG (L)	3.03 × 10^−3^	1.83 × 10^−3^	3.05 × 10^−3^
IFG (R)	8.73 × 10^−4^	1.62 × 10^−3^	8.75 × 10^−4^
Occipital lobe	1.26 × 10^−3^	1.12 × 10^−3^	1.32 × 10^−3^
Parietal lobe	9.47 × 10^−4^	9.72 × 10^−4^	9.50 × 10^−4^
Temporal lobe	1.85 × 10^−3^	1.44 × 10^−3^	1.87 × 10^−3^
**DEEP STRUCTURE**			
Accumbens (L)	2.33 × 10^−3^	1.46 × 10^−3^	2.34 × 10^−3^
Accumbens (R)	1.74 × 10^−3^	1.42 × 10^−3^	1.75 × 10^−3^
Amygdala	2.37 × 10^−3^	1.95 × 10^−3^	2.39 × 10^−3^
Caudate nucleus	1.70 × 10^−3^	1.26 × 10^−3^	1.71 × 10^−3^
Cerebellar GM	1.43 × 10^−3^	1.40 × 10^−3^	1.46 × 10^−3^
Hippocampus	2.01 × 10^−3^	1.52 × 10^−3^	2.03 × 10^−3^
Globus pallidus	1.80 × 10^−3^	1.32 × 10^−3^	1.82 × 10^−3^
Putamen	2.06 × 10^−3^	1.23 × 10^−3^	2.07 × 10^−3^
Thalamus	1.42 × 10^−3^	1.48 × 10^−3^	1.43 × 10^−3^

*The optimal weighting **X** is compared with a symmetric pattern (FPz-Oz) and a pattern found using the 2 greatest weights in **X** (F7-Oz). Median values in the targeted region are highlighted in green shading*.

A test performed using the F7-Oz pattern as an initial point for the procedure resulted in no progress toward the final solution. Interestingly, the first-order optimality measure found using F7-Oz was 3.5 × 10^−3^, larger than that found for the final value of**X** for Problem 2, which was around 2 × 10^−3^.

### Problem 3

**Figure 7:**
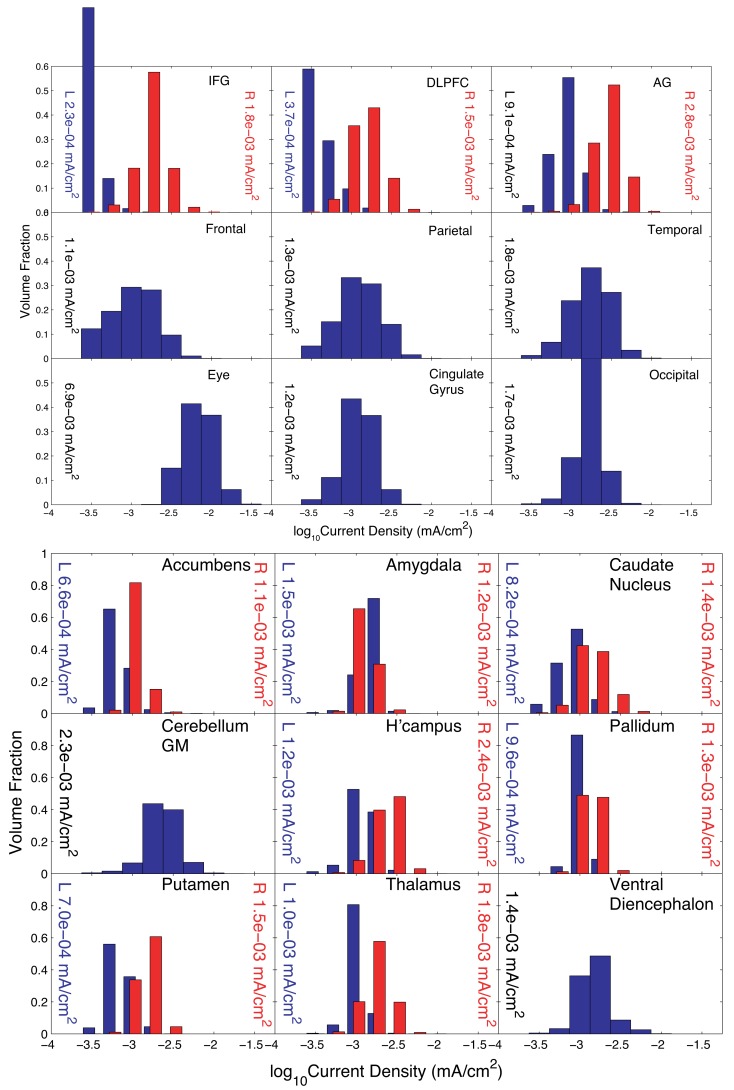
**Distribution of current densities in (top) peripheral cortical structures and (bottom) deep brain structures for Problem 3**. Median values in each structure obtained using the optimal solution **X** are shown within each graph of the figure. IFG, DLPFC, and AG refer to the inferior frontal gyrus (IFG), dorsolateral prefrontal cortex (DLPFC), and angular gyrus (AG), respectively. For the IFG, DLPFC, and AG, current density distributions are shown separately for left (blue) and right (red) structures, with median values shown on either side of each plot.

The pattern found when the IFG was specified as the “avoid” region was biased toward electrodes on the right side of the head, as expected. Execution of Problem 3 was terminated because the step size decreased below threshold. Results for the normalized optimized pattern are shown in Figure 7 for peripheral and deep structures. Table 4 shows median values in different structures for this pattern and for a 2-electrode pattern found by combining the electrodes that had the two largest magnitude weights in **X**–C4 and FPz. Current densities found in the right cortex were generally larger than those in the left cortex or deep brain structures. Median current densities in the eye for this case were larger than in Problem 2 (around 7 × 10^−2^ mA/cm^2^), and therefore phosphene generation would be highly likely with this configuration.

**Table 4:** **Median current density values found in different tissues and structures for Problem 3**.

	**X**	C4-FPz
	mA/cm^2^	mA/cm^2^
**TISSUE**		
Blood	5.31 × 10^−4^	4.67 × 10^−3^
Cancelous bone	1.66 × 10^−3^	1.31 × 10^−3^
Cortical bone	7.18 × 10^−4^	3.42 × 10^−4^
CSF	1.00 × 10^−2^	9.91 × 10^−3^
Fat	2.41 × 10^−3^	1.25 × 10^−3^
Gray matter	1.48 × 10^−3^	1.09 × 10^−3^
Muscle	7.79 × 10^−3^	3.01 × 10^−3^
Sclera	2.90 × 10^−3^	4.93 × 10^−3^
Skin	2.46 × 10^−2^	1.58 × 10^−2^
White matter	3.09 × 10^−3^	2.75 × 10^−3^
**CORTICAL STRUCTURE**		
AG (L)	9.10 × 10^−4^	5.48 × 10^−4^
AG (R)	2.85 × 10^−3^	2.55 × 10^−3^
Cingulate	1.24 × 10^−3^	1.36 × 10^−3^
DLPFC (L)	3.67 × 10^−4^	1.67 × 10^−3^
DLPFC (R)	1.46 × 10^−3^	2.82 × 10^−3^
Frontal lobe	6.89 × 10^−3^	1.94 × 10^−3^
IFG (L)	2.28 × 10^−4^	1.43 × 10^−3^
IFG (R)	1.76 × 10^−3^	2.11 × 10^−3^
Occipital lobe	1.69 × 10^−3^	6.92 × 10^−4^
Parietal lobe	1.26 × 10^−3^	1.03 × 10^−3^
Temporal lobe	1.79 × 10^−3^	7.06 × 10^−4^
**DEEP STRUCTURE**		
Accumbens (L)	6.56 × 10^−4^	1.00 × 10^−3^
Accumbens (R)	1.11 × 10^−3^	1.36 × 10^−4^
Amygdala	1.37 × 10^−3^	1.19 × 10^−3^
Caudate nucleus	1.04 × 10^−3^	1.12 × 10^−3^
Cerebellum GM	2.31 × 10^−3^	7.04 × 10^−4^
Hippocampus	1.57 × 10^−3^	7.85 × 10^−4^
Globus pallidus	1.16 × 10^−3^	8.97 × 10^−4^
Putamen	1.12 × 10^−3^	9.93 × 10^−4^
Thalamus	1.27 × 10^−3^	9.91 × 10^−4^

*The optimal weighting**X** is compared with a pattern found using the 2 greatest weights in X (C4-Pz). Median values in targeted and avoided regions are highlighted in green and red shading, respectively*.

### Use of fewer than 19 electrodes

Results obtained by the optimization, with approximate normalized “optimal” patterns created using the 2-, 4-, and 6-highest magnitude current electrodes are shown in Table 5, now comparing target and avoid regions for each pattern. In this test, if the sum of currents from the set of electrodes was found to be non-zero (contrary to constraint 1), we assumed that remaining current flowed to an extracranial electrode. These electrode patterns resulted in distributions in the target or avoid structures being of the same magnitude as those found using the full 19-electrode montage.

**Table 5:** **Comparison of effects of using 2-, 4- and 6-electrode patterns based on optimal current patterns**.

	**X**2	**X**4	**X**6	**X**19
	mA/cm^2^	mA/cm^2^	mA/cm^2^	mA/cm^2^
**PROBLEM 1**				
IFG (L)	2.19 × 10^−3^	1.77 × 10^−3^	1.46 × 10^−3^	8.26 × 10^−4^
IFG (R)	1.25 × 10^−3^	1.33 × 10^−3^	1.24 × 10^−3^	2.63 × 10^−4^
Accumbens (L)	1.55 × 10^−3^	1.41 × 10^−3^	1.22 × 10^−3^	9.74 × 10^−5^
Accumbens (R)	1.41 × 10^−3^	1.31 × 10^−3^	1.13 × 10^−3^	2.19 × 10^−4^
Skin maximum	3.17 × 10^−1^	2.24 × 10^−1^	1.80 × 10^−1^	2.41 × 10^−1^
**PROBLEM 2**				
IFG (L)	3.02 × 10^−3^	3.05 × 10^−3^	–	3.03 × 10^−3^
IFG (R)	9.06 × 10^−4^	8.75 × 10^−4^	–	8.73 × 10^−4^
Accumbens (L)	2.35 × 10^−3^	2.34 × 10^−3^	–	2.33 × 10^−3^
Accumbens (R)	1.77 × 10^−3^	1.75 × 10^−3^	–	1.74 × 10^−3^
Skin maximum	4.26 × 10^−1^	4.24 × 10^−1^	–	4.18 × 10^−1^
**PROBLEM 3**				
IFG (L)	8.25 × 10^−4^	9.50 × 10^−4^	6.97 × 10^−4^	2.28 × 10^−4^
IFG (R)	1.91 × 10^−3^	1.38 × 10^−3^	1.03 × 10^−3^	1.76 × 10^−3^
Accumbens (L)	1.15 × 10^−3^	9.96 × 10^−4^	7.59 × 10^−4^	6.56 × 10^−4^
Accumbens (R)	1.23 × 10^−3^	9.90 × 10^−4^	7.53 × 10^−4^	1.11 × 10^−4^
Skin maximum	2.34 × 10^−1^	1.32 × 10^−1^	9.71 × 10^−2^	2.31 × 10^−1^

*Medians in targeted and avoided regions are highlighted in green and red shading, respectively. Maximum skin current densities are also shown for each pattern*.

## Discussion

The solution of problem 1 demonstrates how an optimization approach might be used to allow more efficient and precise targeting of tDCS currents to nominated brain regions and enable steering of current away from other specified areas in individual subjects. The solution we found for this problem successfully directed current away from the accumbens (producing a bilateral median current density of 9.74 × 10^−5^ mA/cm^2^) and producing a median current density in the IFG of 8.26 × 10^−4^ mA/cm^2^ in the IFG target. By comparison, the two alternative current patterns, F3-RS and F3-P3, although producing larger current densities in the IFG, both produced median current densities in the accumbens that were at least a factor of 10 larger. The ability to selectively deliver current to different structures may therefore facilitate experiments relating to the structures and mechanisms involved in tDCS effects, particularly when implemented using subject-specific models. Further, use of distributed (i.e., more than two electrodes) current patterns may reduce skin currents and the likelihood of peripheral nerve stimulation and therefore provide a safety benefit over other patterns.

### Use of fewer electrodes

It may also be that patterns using fewer electrodes, based on these “optimal” designs, can be achieved, as demonstrated in Section “Use of Fewer Than 19 Electrodes.” These patterns could be implemented by coupling several current generators together. Use of a selection of higher weighted electrodes in combination with a single extracranial electrode might provide a practical method of implementing computed patterns.

### Safety considerations

The maximal skin currents shown in Table 5 were reduced as more electrodes were incorporated. Therefore, use of more electrodes may make it possible to apply a larger total current and achieve some current steering without causing peripheral nerve stimulation. The nominal current density value thought to produce peripheral nerve stimulation is about 0.1 mA/cm^2^ (Reilly, 1998). Note that in all but one case shown in Table 5, the predicted maximum skin current densities were above this limit. However, these current densities were observed in very small volumes near electrodes, and it is unclear whether these patterns would actually result in a subject’s perception of the current. In the two problems targeting deep structures, we observed median eye currents of the order of 0.1 mA/cm^2^. This prediction implies that phosphene generation is likely using these patterns. Use of the eye as an “avoid” region might produce more acceptable patterns.

### Use of more constraints

The problems we have considered here involve a fixed amount of current applied to the head. This current must flow somewhere. Use of “avoid” constraints may result in large currents being observed in areas that are neither avoided nor target regions, such as those found in right peripheral cortical regions in Problem 3. This issue will obviously be more prevalent as more avoided regions are chosen and will depend on the relative geometry of electrodes, avoided regions, and target regions. We expect that it may not be possible to solve some over constrained optimization tasks, or to find a feasible starting point.

A corollary finding is that these observations may be beneficial and provide alternatives to previous stimulation protocols. For example, the median *J* found in the left IFG by Problem 2 was larger than that found using the F3-RS current pattern, which has been presumed appropriate for stimulating this area. If applying a large current to the left IFG is the only requirement, then a pattern similar to that found in Problem 2 might also be considered to stimulate the IFG of a similar subject.

### Optimality

The results we have found have satisfied the requirements specified to the optimization algorithm, with some exceptions. However, there is no guarantee that the solution is a global optimum or even unique. A trivial demonstration of the non-uniqueness of solutions is that exactly the same current densities as any candidate weighting, **X**, will be produced by −**X**, since most constraints and objective function are based solely on current density magnitude. This lack of uniqueness could be resolved by introducing a constraint on a single electrode, *i*, that restricted its coefficient, *X*_i_, to be either less than or greater than zero.

The optimality measure produced by the algorithm, a numerical measure of the gradient of the objective function at each iteration, was found to be less than 10^−3^ for solution of Problem 1. We know that our gradient estimation is not exact, but this value should provide some indication of the landscape of the objective function. Even if gradient estimation is exact, finding an optimality measure that suggests the objective function is at or near an extreme value does not guarantee that the solution attained is a global minimum.

Solutions in Problems 2 and 3 produced optimality measures of around 2 and 3 × 10^−3^, respectively. Solutions in these two problems took many more iterations to produce than in Problem 1, and solution of Problem 2 was terminated because the algorithm required more than 100 iterations. Very similar results to the optimal solution (**X**) to Problem 2 were found using its two principal electrodes, and, in fact, *J* values in the target structure were slightly larger when the two principal electrodes were used. It is possible that the F7-Oz solution is very close to the optimum solution for this Problem, and with this subject model. This finding may also suggest that solutions targeting of deep structures may not be unique, and that there are other possible configurations that satisfy the problem specification.

## Conclusion

We demonstrated that use of a finite element model of the head, in conjunction with a non-linear optimization procedure, could result in current steering both away from and toward different structures. We found that it was possible to direct current to the left IFG while avoiding the accumbens region; to target current on the basal ganglia exclusively; and to avoid the left IFG while targeting basal ganglia. When deep structures were targeted, it was not possible to avoid delivering current to peripheral cortical regions. Further, use of this methodology revealed asymmetry in structures that may not have easily been found using other strategies. We believe that this or a similar method of optimization may prove useful in further studies of tDCS.

## Conflict of Interest Statement

The authors declare that the research was conducted in the absence of any commercial or financial relationships that could be construed as a potential conflict of interest.

## References

[B1] Adrian D. J. (1977). Auditory and visual sensations stimulated by low-frequency electric currents. Radio Sci. 12, 243–250 10.1029/RS012i06Sp00243

[B2] Akhtari M. Bryant H. C. Mamelak A. N. Flynn E. R. Heller L. Shih J. J. (2002). Conductivities of three-layer live human skull. Brain Topogr. 14, 151–167 10.1023/A:1014590923185 12002346

[B3] Akhtari M. Bryant H. C. Mamelak A. N. Heller L. Shih J. J. Mandelkern M. (2000). Conductivities of three-layer human skull. Brain Topogr. 13, 29–42 10.1023/A:1007882102297 11073092

[B4] Baumann S. B. Wozny D. R. Kelly S. K. Meno F. M. (1997). The electrical conductivity of human cerebrospinal fluid at body temperature. IEEE Trans. Biomed. Eng. 44, 220–223 10.1109/10.554770 9216137

[B5] Bonnans J. F. Gilbert J. C. Lemarechal C. Sagastizabal C. (2006). Numerical Optimization – Theoretical and Practical Aspects, 2nd Edn Berlin: Springer Verlag

[B6] Brunelin J. Mondino M. Gassab L. Haesebaert F. Gaha L. Suaud-Chagny M. (2012). Examining transcranial direct-current stimulation (tDCS) as a treatment for hallucinations in schizophrenia. Am. J. Psychiatry 169, 719–724 22581236 10.1176/appi.ajp.2012.11071091

[B7] Brunoni A. Nitsche M. A. Bolognini N. Bikson M. Wagner T. Merabet L. (2012). Clinical research with transcranial direct current stimulation (tDCS): challenges and future directions. Brain Stimul. 5, 175–195 10.1016/j.brs.2011.03.002 22037126 PMC3270156

[B8] Cogiamanian F. Marceglia S. Ardolino G. Barbieri S. Priori A. (2007). Improved isometric force endurance after transcranial direct current stimulation over the human motor cortical areas. Eur. J. Neurosci. 26, 242–249 10.1111/j.1460-9568.2007.05633.x 17614951

[B9] Datta A. Bansal V. Diaz J. Patel J. Reato D. Bikson M. (2009). Gyri-precise head model of transcranial direct current stimulation: improved spatial focality using a ring electrode versus conventional rectangular pad. Brain Stimulat. 2, 201–207 10.1016/j.brs.2009.03.005 PMC2790295 20648973

[B10] Dmochowski J. P. Datta A. Bikson M. Su Y. Parra L. C. (2011). Optimized multi-electrode stimulation increases focality and intensity at target. J. Neural Eng. 8, 046011 10.1088/1741-2560/8/4/046011 21659696

[B11] Elmer S. Burkard M. Renz B. Meyer M. Jancke L. (2009). Direct current induced short-term modulation of the left dorsolateral prefrontal cortex while learning auditory presented nouns. Behav. Brain Funct. 5, 29 10.1186/1744-9081-5-29 19604352 PMC2719658

[B12] Fregni F. Boggio P. S. Nitsche M. A. Bermpohl F. Antal A. Feredoes E. (2005). Anodal transcranial direct current stimulation of prefrontal cortex enhances working memory. Exp. Brain Res. 166, 23–30 10.1007/s00221-005-2334-6 15999258

[B13] Gabriel C. Gabriel S. Corthout E. (1996). The dielectric properties of biological tissues: I. Literature survey. Phys. Med. Biol. 41, 2231–2249 10.1088/0031-9155/41/11/002 8938024

[B14] Geddes L. Baker L. E. (1967). The specific resistance of biological materials: a compendium of data for the biomedical engineer and physiologist. Med. Biol. Eng. Comput. 5, 271–293 10.1007/BF02474537 6068939

[B15] Holdefer R. N. Sadleir R. J. Russell M. J. (2006). Predicted current densities in the brain during transcranial electrical stimulation. Clin. Neurophysiol. 117, 1388–1397 10.1016/j.clinph.2006.02.020 16644273 PMC2426751

[B16] Im C.-H. Jung H.-H. Choi J.-D. Lee S. Y. Jung K.-Y. (2008). Determination of optimal electrode positions for transcranial direct current stimulation (tDCS). Phys. Med. Biol. 53, N219–N225 10.1088/0031-9155/53/11/N03 18490807

[B17] Kirkpatrick S. Gelatt C. D. Vecchi M. P. (1983). Optimization by simulated annealing. Science 220, 671–680 10.1126/science.220.4598.671 17813860

[B18] Limpert E. Stahel W. A. Abbt M. (2001). Log-normal distributions across the sciences: keys and clues. Bioscience 51, 341–352 10.1641/0006-3568(2001)051[0341:LNDATS]2.0.CO;2

[B19] Loo C. K. Alonzo A. Martin D. Mitchell P. B. Galvez V. Sachdev P. (2012). Transcranial direct current stimulation for depression: 3-week, randomised, sham-controlled trial. Br. J. Psychiatry 200, 52–59 10.1192/bjp.bp.111.097634 22215866

[B20] Monti A. Cogiamanian F. Marceglia S. Ferrucci R. Marmeli F. Mrackic-Sposta S. (2008). Improved naming after transcranial direct current stimulation in aphasia. J. Neurol. Neurosurg. Psychiatr. 79, 451–453 10.1136/jnnp.2007.135277 18096677

[B21] Nitsche M. A. Cohen L. G. Wassermann E. M. Priori A. Lang N. Antal A. (2008). Transcranial direct current stimulation: state of the art 2008. Brain Stimulat. 1, 206–223 10.1016/j.brs.2008.06.004 20633386

[B22] Nitsche M. A. Paulus W. (2000). Excitability changes induced in the human motor cortex by weak transcranial direct current stimulation. J. Physiol. 527, 633–639 10.1111/j.1469-7793.2000.t01-1-00633.x 10990547 PMC2270099

[B23] Park J. H. Hong S. B. Kim D.-W. Suh M. Im C.-H. (2011). A novel array-ype transcranial direct current stimulation (tDCS) system for accurate focusing on targeted brain areas. IEEE Trans. Magn. 47, 882–885 10.1109/TMAG.2011.2134077

[B24] Priori A. Mameli F. Cogiamanian F. Marceglia S. Tiriticco M. Mrackic-Sposta S. (2008). Lie-specific involvement of dorsolateral prefrontal cortex in deception. Cereb. Cortex 18, 451–455 10.1093/cercor/bhm088 17584853

[B25] Reilly J. P. (1998). Applied Bioelectricity: From Electrical Stimulation to Electropathology. New York: Springer

[B26] Rubin M. Safdieh J. E. (2007). Netter’s Concise Neuroanatomy. Philadelphia: Saunders Elsevier

[B27] Sadleir R. J. Argibay A. (2007). Modeling skull electrical properties. Ann. Biomed. Eng. 35, 1699–1712 10.1007/s10439-007-9343-5 17629793 PMC2496996

[B28] Sadleir R. J. Vannorsdall T. D. Schretlen D. J. Gordon B. (2010). Transcranial direct current stimulation (tDCS) in a realistic head model. Neuroimage 51, 1310–1318 10.1016/j.neuroimage.2010.03.052 20350607

[B29] Scott G. C. Joy M. L. G. Armstrong R. L. Henkelman R. M. (1991). Measurement of nonuniform current density by magnetic resonance. IEEE Trans. Med. Imaging 10, 362–374 10.1109/42.97586 18222838

[B30] Wagner T. Fregni F. Fecteau S. Grodzinsky A. Zahn M. Pascual-Leone A. (2007). Transcranial direct current stimulation: a computer-based human model study. Neuroimage 35, 1113–1124 10.1016/j.neuroimage.2007.01.027 17337213

[B31] Wakana S. Jiang H. Nagae-Poetscher L. M. Van Zijl P. C. M. Mori S. (2004). Fiber tract-based atlas of human white matter anatomy. Radiology 230, 77–87 10.1148/radiol.2301021640 14645885

[B32] Waltz R. A. Morales J. L. Nocedal J. Orban D. (2006). An interior algorithm for nonlinear optimization that combines line search and trust region steps. Math. Programming 107, 391–408 10.1007/s10107-004-0560-5

